# Chilblain

**Published:** 1861-03

**Authors:** 


### Chilblain.

Dr. James E. Garrison, of Philadelphia, publishes in the *Reporter*, of the 19th January, the following formula for chilblain, which he has used with remarkable success:

| | |
|---|---|
| ℞.—Ferri per sulph. (Mousel's solution), | say, f ʒ i. |
| Tinct. Iod., Lin Saponis, } aa | say, f ℥ i. |

He advises that "the quantity of each article to be directed, of course, by the indications of each particular case. Before applying the liniment, which may be painted with a brush, or applied by wrapping up the toes with saturated raw cotton, the feet are to be held for two or three minutes in cold water, and next rubbed into a glow with a crash towel."

